# A Clinicopathologic Study of Excised Conjunctival Lesions

**DOI:** 10.4103/0974-9233.75886

**Published:** 2011

**Authors:** Laila Hassan Mohamad Elshazly

**Affiliations:** Department of Ocular and Pathology, Memorial Institute of Ophthalmology, Cairo, Egypt

**Keywords:** Conjunctiva, Hemangioma, Nevus, Ocular Surface Squamous Neoplasia, Papilloma

## Abstract

**Purpose:**

This study was aimed at to determine the frequency of excised conjunctival lesions in a patient population treated over a 10-year period.

**Materials and Methods:**

The data of all excised conjunctival lesions with tissue diagnoses from 1998 to 2008 in the pathology department were analyzed.

**Results:**

The patient group comprised 192 conjunctival specimens; 106 (55.2%) obtained from male patients and 86 (44.8 %) from female patients. The age range was 75 years with a mean age of 27.07 ± 17 years. The most frequent excised lesions were pyogenic granulomas, which represented 30.7% (59 cases). Pigmented epithelial tumors were the second most common benign conjunctival lesions (44 cases, 22.9%). Out of these cases, compound nevus represented 86.4% (38 cases) and junctional nevus represented 6.8% (3 cases). Primary acquired melanosis and subepithelial nevus were reported in two cases (4.5%) and one case (2.3%), respectively. Cystic lesions represented 12% (23 cases). These were mostly ductal retention cysts in 16 cases (70%), occupying the fornix in eight cases. Ocular surface squamous neoplasia (OSSN) was detected in 21 cases (10.9%) significantly affecting an older age group as compared to other lesions (mean age 45.9 ± 16.7). Other less frequent lesions included papilloma (10 cases, 2.5%), dermolipoma (8 cases, 4.2%), solid dermoid (3 cases, 1.6%), hemangioma (15 cases, 7.8%), and benign reactive lymphoid hyperplasia (four cases, 2.1%).

**Conclusion:**

Benign lesions were the most frequent histologically diagnosed conjunctival lesions. The true malignant lesions were lower than what has been described in many reports. The significant proportion of precancerous OSSN can be attributed to sun exposure and ultraviolet light in Egypt.

## INTRODUCTION

Excised lesions of the conjunctiva include a wide spectrum of conditions ranging from benign lesions, such as pterygium, solid dermoid, nevus, papilloma, hemangioma and pyogenic granuloma, to precancerous ocular surface squamous neoplasia (OSSN) and aggressive malignancies, such as malignant melanoma, squamous cell carcinoma, or Kaposi’s sarcoma.^1^ The differentiation of these lesions is based on the patient’s history, clinical, and histopathological features of the lesion. The conjunctiva is readily visible, so related lesions that occur in the conjunctiva are generally recognized at a relatively early stage.

There is a relative paucity of large published series documenting conjunctival lesions. A review of a large series of conjunctival biopsy specimens from an adults US population documented the following distribution: inflammatory/degenerative lesions (12%); benign epithelial (2%); pigmented (53%); premalignant and malignant epithelial (11%); lymphoid (8%); miscellaneous (12%); and congenital lesions (2%). 2 A similar series in the Indian population, reported 46% of the lesions were of epithelial origin (benign, premalignant, and malignant neoplasm). The remaining lesions included miscellaneous lesions (22%), degenerative lesions (14%), melanocytic tumors (12%), and lymphoid tumors (6%).^3^ Squamous cell carcinoma occurred in 20% followed by chronic non-specific inflammation (12%), pterygium (10%), squamous papilloma (8%), and OSSN (8%).^3^

The aim of this retrospective study was to determine the frequency of the most commonly excised conjunctival lesions in a cohort of subjects with histopathologically confirmed diagnoses over a 10-year period.

## MATERIALS AND METHODS

The data on conjunctival masses diagnosed in the pathology department were retrospectively analyzed from 1998 to 2008. Pterygia were excluded due to nonreferral of the majority of these lesions. Age, sex distribution of patients, and location of lesions were evaluated according to hospital pathological referral records. Hematoxylin and eosin stained sections from each biopsy specimen were examined. The final diagnosis in each case was established by a combination of history, ocular findings, and histopathology.

The Students t-test and analysis of variance (ANOVA) test were used to analyze continuous variables such as age whereas the Chi-square (χ2) test was used to analyze categorical variables such as sex and site of lesion. A *P*-value less than 0.05 was considered statistically significant.

## RESULTS

A total of 192 conjunctival specimens were examined in the pathology department in the decade spanning 1998 to 2008. Of this specimens, 106 (55.2%) were from males and 86 (44.8%) for females. The conjunctival specimens were referred to the pathology department due to suspicion of a conjunctival neoplasm because of rapid growth or for cosmetic reasons. The mean age of the cohort was 27.07 ± 17 years (range, 1.5–77 years), and the mode was 20 years (*P* = 0.0001, ANOVA). There was no significant difference in sex distribution (*P* = 0.149, Chi-square test). The distribution of the lesions is presented in Table 1. Differences in mean age between the lesions are shown in Table 2.

**Table 1:** Distribution of excised conjunctival lesions in relation to the age and sex of the cohort

	Number (%)	Age (years) (Mean + SD)	Sex male (%):female (%)
Pyogenic granuloma	59 (30.7)	30.99 ± 17.2	34 (57.6):25 (42.4)
Nevus	44 (22.9)	19.48 ± 10.57	21 (47.7):23 (52.3)
Cyst	23 (12)	28.26 ± 16.57	13 (56.5):10 (43.5)
OSSN	21 (10.9)	45.9 ± 16.7*	9 (40.9):12 (54.5)
Hemangioma	15 (7.8)	16.8 + 9.15	9 (60):6 (40)
Papilloma	10 (5.2)	25.9 ± 21.1	5 (50):5 (50)
Dermolipoma	8 (4.2)	18.63 ± 8.16	4 (50):4 (50)
Solid dermoid	3 (1.6)	5.0 ± 1.5	2:1
BLH	4 (2.1)	19.75 ± 7.59	3:1
Nonspecific inflammation	4 (2.1)	28.25 ± 19.55	3:1
Herniated orbital fat	1 (0.5)	30	0:1
Total	192	27.07 ± 17.02	106 (55.2):86
			(44.8)
*P* value		0.0001*	0.149†

OSSN - Ocular surface squamous neoplasia; BLH - benign lymphoid hyperplasia

* Significant at *P* < 0.05 (ANOVA),

† Chi test

**Table 2:** *P* values of the *t*-test of the differences in mean age between conjunctival lesions

Disease	Pyogenic granuloma	Nevus	Cystic	OSSN	Hemangioma	Papilloma	Dermolipoma	Solid dermoid
Pyogenic granuloma	-	0.0001*	0.516	0.001*	0.003*	0.41	0.05	0.0001*
Nevus	0.0001*	-	0.01*	0.0001*	0.019*	0.19	0.169	0.023*
Cystic	0.516	0.013*	-	0.001*	0.02*	0.732	0.128	0.025*
OSSN	0.001*	0.0001*	0.001*	-	0.0001*	0.008*	0.0001*	0.0001*
Hemangioma	0.003*	0.019*		0.0001*	-	0.056	0.642	0.045
Papilloma	0.41	0.19	0.732	0.008*	0.056	-	0.373	0.125
Dermolipoma	0.05	0.169	0.128	0.0001*	0.642	0.373	-	0.021*
Solid dermoid	0.0001*	0.023*	0.025*	0.0001*	0.045	0.125	0.021*	-
Lymphoid hyperplasia	0.20	0.96	0.329	0.006*	0.563	0.588	0.823	0.023*

OSSN - Ocular surface squamous neoplasia

* Significant difference of the age between lesions with the *t*-test (*P* < 0.05)

**Figure 1:**
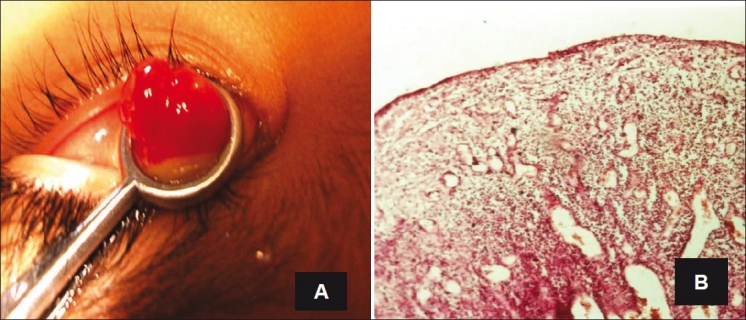
A case of pedunculated pyogenic granuloma with a lobulated surface involving the palpebral conjunctiva after previous chalazion excision (A). Histopathologically, it had dilated and congested capillaries separated by fibrotic stroma with inflammatory cells infiltration (H and E, ×25) (B)

The most frequent lesions were pyogenic granulomas in 59 cases (30.7%) with correct clinical diagnoses in 55 cases. The four clinical misdiagnoses were carcinoma in situ, hemangioma, papilloma, and conjunctival cyst. Pyogenic granulomas diagnosed on the palpebral conjunctiva generally developed following chalazion excision (12 cases, 20.3%). Pyogenic granulomas of the bulbar conjunctiva were related to pterygium excision in 12 cases (20.3%), squint surgery in 1 case, and trauma in 3 cases (5.1%). No specific etiology was reported in 31 cases (52.5%). Pyogenic granulomas were clinically described as pedunculated or sessile soft vegetation of excessive granulation tissue. Histopathologically, these granulomas had dilated and congested capillaries separated by fibrotic stroma with variable grades of inflammatory cells infiltration [Figure 1]. Pyogenic granulomas significantly affected middle-aged patients with statistically significant differences from nevus, OSSN, hemangioma, and solid dermoid (*P* = 0.0001, 0.001, 0.003, and 0.0001, respectively; *t*-test) [Table 2].

**Figure 2:**
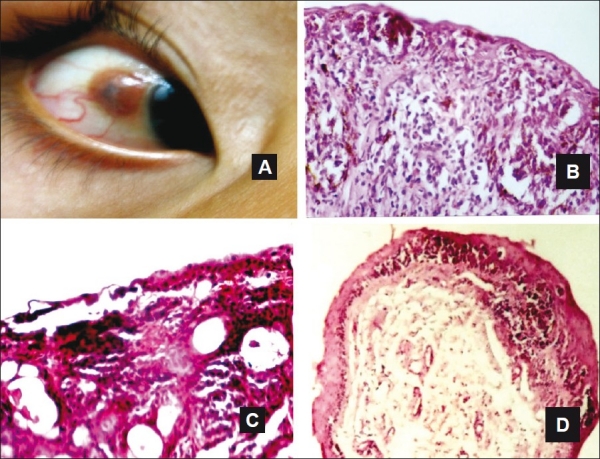
A case of compound nevus involving the bulbar conjunctiva (A). Nevus cells with junctional activity in the epidermodermal junction and the dermis with excessive chronic inflammatory cells infiltration (H and E, ×100) (B). Photomicrographs of; cystic nevus (C), junctional nevus with nevus cells at the epidermodermal junction (D) (C, D H and E, ×25)

Melanocytic nevi were the second most common benign conjunctival lesion (44 cases, 22.9%). These nevi occurred most commonly on the bulbar conjunctiva (65.9%, 29 cases), followed by the limbus (27.3%, 12 cases), the fornix, the caruncle, and outer canthus (one case each). Nevus excision was mostly due to rapid growth and/or cosmesis. Histopathologically, 38 cases (86.4%) were diagnosed as compound nevi with nests of nevus cells with junctional activity in the epidermodermal junction and the dermis. Of these, 18 cases showed excessive chronic inflammatory cells infiltration with numerous eosinophils, nine of them were accompanied with cystic spaces. Purely cystic compound nevi were detected in five cases [Figures 2A–C]. Most of the inflamed and cystic nevi were juxtalimbal. Three subjects (6.8%), aged 10, 16, and 20 years, with lesion history since birth, were diagnosed with junctional nevi and nests of nevus cells in the epidermodermal junction [Figure 2D]. Primary acquired melanosis (PAM) without atypia occurred in two cases (4.5%). Subepithelial nevus was diagnosed in one case (2.3%).

Conjunctival nevi occurred in younger patients with a mean age of 19.48 ± 10.57 years. There were significant differences from pyogenic granuloma, cystic lesions, OSSN, hemangioma, and solid dermoid (*P* = 0.0001, 0.01, 0.0001, 0.019, and 0.023, respectively, *t*-test).

**Figure 3:**
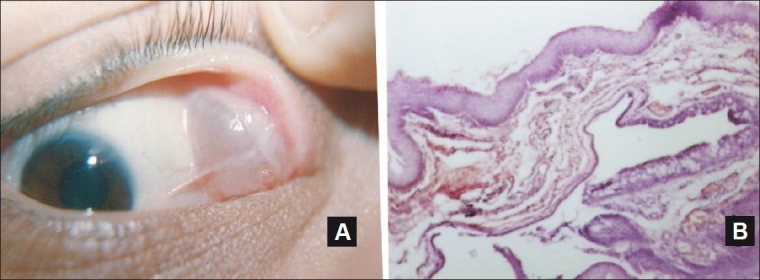
A retention cyst occupying the temporal fornix (A). Photomicrograph showed the cubical cell layer lining of the cyst with area of epithelium rich in goblet cells (H and E, ×25) (B)

There were 23 cystic lesions (12%), 16 (70%) of which were ductal retention cysts of which eight cases (34.8%) were occupying the fornix. Two cases of traumatic blood cysts and two cases of mucous glands retention cysts were located in the bulbar conjunctiva [Figure 3]. Epidermoid cysts were detected in three cases involving the caruncle. Cystic lesions in middle-aged patients occurred with significant difference from nevus, OSSN, hemangioma, and solid dermoid (*P* = 0.013, 0.001, 0.02, and 0.025, respectively, t-test). No significant difference from pyogenic granuloma was noted in middle-aged subjects (*P* = 0.516, t-test) [Table 2].

**Figure 4:**
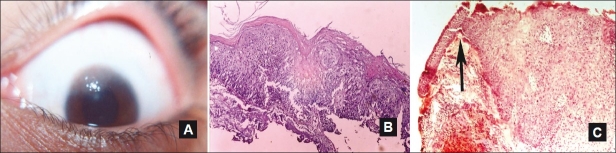
Well-defined limbal OSSN encroaching over the cornea with gelatinous surface (A). Replacement of conjunctival epithelium by dysplastic squamous cells, thickening, and keratinization of superficial cells (atypia) (B). Sudden transition between normal and dysplastic area (arrow) with intact basement membrane (C). (B and C) H and E, ×25)

OSSN was detected in 21 cases (10.9%). The mean age of subjects affected with OSSN was 45.9 ± 16.7 (range from 22 to 77 years) with nine cases were older than 50 years. OSSN affected older patients compared to all other lesions (Table 2, *P* < 0.05, t-test). OSSN was clinically described as sessile, raised, and pink-to-reddish gray in color with irregular or gelatinous surface and well-defined edges. The limbal area was affected in 18 cases (85.7%) which was significantly different from other lesions (*P* = 0.0001, Chi-square test). Feeder vessels were reported in some cases. Histopathological examination revealed partial to full thickness replacement of conjunctival epithelium by dysplastic squamous cells, intact basement membrane, and an abrupt transition between the normal conjunctiva and the dysplastic area [Figure 4]. Although atypia was detected in 17 cases, invasive squamous cell carcinoma was not seen in any of the cases.

Hemangiomas represented 7.8% (15 cases) with mean age of 16.8 ± 9.15 years. It affected younger patients with a significant difference from pyogenic granuloma, nevus, cystic lesions, and OSSN (*P* = 0.003, 0.019, 0.02, and 0.0001, respectively, t-test). Capillary hemangioma was detected in 9 of 15 hemangioma cases. Hemangioendothelioma was diagnosed in five cases and angiofibroma in one case. Hemangiomas occurred most commonly on the bulbar conjunctiva (10 cases).

Papilloma occurred in 10 cases (5.2%) involving the lacrimal punctum (three cases), caruncle (three cases), limbal (two cases), bulbar (one case), and palpebra (one case). It was clinically described as a pedunculated, papillary reddish lesion. Sessile papilloma was seen in limbal and palpebral cases. Histopathologically, it had a vascular connective tissue core lined by a hyperplastic epithelium. It significantly affected the younger and middle-aged patients with no significant difference from pyogenic granuloma, nevus, cystic lesions, and hemangiomas (*P* = 0.41, 0.19, 0.732, and 0.056, respectively, t-test) [Table 2].

Eight cases (4.2%) of dermolipoma were described as yellowish subconjunctival mass involving the outer canthus with no palpable posterior margin. Dermolipomas were partially excised in most cases for cosmetic reasons due to posterior orbital extension. Histologically, there was excessive fat tissue with associated retention ductal cysts in some cases. Solid limbal dermoids were excised in three cases (1.6%) due to rapid growth that encroached the cornea and for cosmetic reasons. They were clinically described as solid elevated masses embedded in the superficial sclera and/or cornea on the inferior temporal globe or temporal limbus, with occasional fine protruding hairs. Histologic composition was fibrous tissue and occasional hair with sebaceous glands below the conjunctival epithelium.

Benign lymphocytic hyperplasia and chronic nonspecific inflammation occurred in four cases each, with a mean age of 19.75 ± 7.59 years and 28.25 ± 19.55 years, respectively. The benign lymphocytic hyperplasia was described as a salmon-pink conjunctival mass involving the caruncle or the fornix. Histologically, it had diffuse infiltration of the subepithelial tissue with mature lymphocytes and few lymphoid follicles. There was no accompanying systemic affection or regional lymphadenopathy as reported by a medical doctor. Patients’ records included physical examination, complete blood cell count, erythrocyte sedimentation rate, and orbital computed tomography. Cases of chronic nonspecific inflammation showed multiple cell types such as lymphocytes, plasma cells, and occasional eosinophils.

With respect to site of occurrence for lesions, the bulbar conjunctiva was the most common site with 82 cases (42.7%), followed by the limbal area with 41 cases (21.4%), the palpebral conjunctiva with 30 cases (15.6%), and the caruncle with 16 cases (8.3%). There was a significant difference in localization between excised lesions (*P* = 0.0001, Chi-square test). The mean age for limbal, palpebral, and caruncular lesions was not significantly different (*P* = 0.063, ANOVA). There was a significant difference in mean age between limbal and bulbar lesions (*P* = 0.0001, ANOVA). Table 3 shows the distribution of excised lesions according to age and site.

**Table 3:** Distribution of conjunctival lesions according to site and age

Site	Number (%)	Age(mean ± SD)
Limbal	41 (21.4)	35.927 ± 20.34
Bulbar	82 (42.7)	22.798 ± 14.12
Palpebral	30 (15.6)	30.4 ± 14.86
Caruncle	16 (8.3)	25.125 ± 19.24
Fornix	11 (5.7)	22.727 ± 10.98
Punctum	3 (1.6)	45.33 ± 28.59
Outer canthus	9 (4.7)	17.11 ± 8.88
Total	192	27.07 ± 17.021
*P* value	0.0001*	0.0001†

* Significant (Chi-test),

† Significant (ANOVA)

## DISCUSSION

This analysis of 192 conjunctival lesions can be used a representative sample of the Egyptian population. Studies on a large cohort of eyes can also be used for future comparative studies. In this study, the most common benign lesion that was excised was pyogenic granuloma. This contradicts a previous study on US subjects which reported an incidence of 1% for pyogenic granuloma [Table 4].^2^ Pyogenic granulomas were related to cases of chalazion, pterygium, and squint surgery in close to half the cases (47.5%). This outcome is consistent with earlier studies suggesting major trauma, subclinical infections, inflammation, or previous surgery as the major etiologic factor.^4^,^5^ The lack of previous history in 52.5% of our cases would suggest that the lesions arose *de novo* from an unknown stimulus or insult. We did not find statistically significant differences in occurrence site or frequency between sexes which was different from Panda *et al*.^6^ who suggested preponderance among males. The differences in sample size and characteristics may explain this difference between studies.

**Table 4:** Lesion characteristics, frequency, and demographics of previous published studies in the United States^2^, Iran^7^, and India^3^

	Shield *et al*.^2^				Amoli *et al*.^7^			Mondal *et al*.^3^			
	*n* (%)	Age*	Sex M(%):F(%)	Site†	*n* (%)	Age*	Sex M:F	*n* (%)	Age*	SexM:F	Site†
Choristoma	40 (2)	26	15 (38): 25 (62)	Bulbar	25 (5.6)	11		-			
Benign											
Epithelial (papillomas)	38 (2)	46	30 (79): 8 (21%)	Limbal	35 (7.8)	55.3		6 (12)			
Malignant											
SCC	108 (6)	66	146 (81): 35 (19)	Limbal	112(25.1)	58.63		10(20)	>65	12:2	Bulbar and limbal
CIN (OSSN)	71 (4)				25(5.6)	54.1		4(8)			
Actinic keratosis	-				17(3.8)	50.12		-			
BCC	2 (<1)				-	-		-			
Mucoepidermoid carcinoma	-				1(0.2)	65					
Melaocytic (total)	872 (53)	45	394 (45): 478 (55)	Limbal	173(38.7)	22.3		6 (12)			Bulbar and limbal
Nevus	454 (52)				10 (2.2)	40.5		Two cases			
PAM	180 (21)				18 (4)	59.39		One case			
MM	215 (25)				1 (0.2)	29		Three cases			
Secondary acquired melanosis	-										
Vascular (total)	63 (4)	39	36 (57): 27 (43)	Bulbar	23 (5.1)	22.26		2 (4)			Palpebraland bulbar
Pyogenic granuloma	11 (1)										
Hemangiomas	52 (3)										
Cystic	27 (1)							3 (6)			
Fibrous	7 (<1)	39	4 (57): 3 (43)	Bulbarand limbal	2 (0.4)	59					
Neural	1 (<1%)	49	1 (100): 0 (0)	Limbal	1 (0.2)	3		-			
Xanthoma	1 (<1%)	6	1 (100): 0 (0%)	Limbal	1 (0.2)	3		-			
Myxomatous	1 (<1%)	29	0 (0): 1 (100)	Bulbar	-	-		-			
Lipomatous (total)	23 (<1%)	61	13 (57): 10 (43)	Bulbar	-	-			-		
Herniated orbital fat	20 (<1)										
Lacrimal gland	12 (<1%)	57	6 (50): 6 (50)	Fornix	-	-		-			
Lymphoid (total)	128 (8%)	57	64 (50): 64 (50)	Fornix	3 (0.7)	72		3 (6)			Bulbar and fornix and diffuse
Lymphoma								Two cases			
BLH								One case			
Leukemic	3 (<1%)	72	1 (33): 2 (67)	Equal limbal, bulbar, palpebral	-	-		-			
Metastatic	13 (<1%)	54	7 (54): 6 (46)	Bulbar				-			
Secondary	54 (3%)	64	22 (41): 32 (59)	Bulbar				-			
Degenerative and inflammatory simulating tumors	179 (12%)	48	102 (50): 104 (50)	Bulbar				16 (32)			Bulbar
Total	1643		842:801		447	38.7	270:177	50		34:16	

* Age is expressed as mean M:F;male:female

† Site represents the commonest site of affection

SCC - Squamous cell carcinoma; CIN - conjunctival intraepithelial neoplasia or OSSN; BCC - Basal cell carcinoma; MM - Malignant melanoma; BLH - benign lymphoid hyperplasia

In this study, nevi were the second commonly excised tumor, which contradicts the work of Amoli *et al*..^7^ Similar to our study, the prevalence of compound nevi among young patients within the second decade of life with the majority involving the bulbar conjunctiva has been previously reported.^8^,^9^ Rapid growth of compound nevi was attributed to cystic spaces and/or inflammatory cellular infiltration in 23 cases. Eosinophil infiltration suggested a high incidence of associated allergy.^10^,^11^ The growth of the remaining nevi could be explained by nevus cells proliferation. Recently, high levels of mutated B-Raf proto-oncogene serine/threonine-protein kinase (BRAF) gene has been discovered in benign nevi. The mutated BRAF gene may activate the mitogen-activated protein kinase pathway, which stimulates multiple protein kinases that regulate cell proliferation, differentiation, and angiogenesis.^12^

Juxtalimbal PAM without atypia was present in two subjects aged 34 and 40 years with a history of increased pigmentation. The histologic examination did not reveal atypical melanocytes. Previous studies have reported that PAM with atypia carries a 13% risk of transformation to a malignant conjunctival melanoma.^13^ Sun exposure was found to induce DNA damage of superficial epithelial cells with activation of transcription factors that stimulate increased expression of melanin-producing enzymes causing increased pigmentation.^14^ Malignant melanoma was not detected in this study, which emphasizes the rarity of this lesion which has a reported incidence ranging from 0.2 to 0.8 per million in white- or fair-skinned populations.^15^ Additionally benign nevi with mutated *BRAF* infrequently become malignant, suggesting additional genetic insults are necessary for malignancy.^12^

In this study, OSSN was the most frequent tumor in old age with nearly equal sex distribution despite previous report of a fivefold higher incidence among white males.^16^ OSSN occurs in about 0.2–12 cases per 1,000,000 per year with geographic and ethnic variations.^17^ In our study, there were 11 cases younger than 50 years although occurrence of OSSN below 50 years of age is rare with very few cases reported in the literature.^18^ Causative factors that contribute to OSSN include ultraviolet light (UV-B) exposure, ocular trauma, predisposing genetic factors, and HPV human papilloma virus or human immune virus infection.^19^ These premalignant neoplasms can develop into invasive squamous cell carcinoma characterized by increased thickness of epithelial dysplastic changes, invasion into the substantia propria with malignant squamous epithelial cell with keratin-filled epithelial pearl formation in well-differentiated tumors. The tumor then grows slowly, invading nearby tissues including the globe, eyelids, and orbital tissues leading to severe visual loss, loss of the eye, and severe facial deformities.^1^

In this study, four cases of benign reactive lymphoid hyperplasia were detected. Two subjects only 14 years of age and the oldest was 30 years. Three of the subjects were males in accordance with previous reports that suggested higher incidence among males.^20^ We found very few cases of benign lymphoid hyperplasia affecting children documented in the literature.^21^ Reactive lymphoid hyperplasia were considered a gray zone with difficult histopathologic differentiation from malignant lymphoma. Histologically, they consist of small lymphocytes with absent Dutcher bodies (intranuclear eosinophilic inclusions) and limited mitosis within the germinal centers of lymphoid follicles.^1^ Immunophenotyping and immunogenotyping show polyclonal T- and B-cell infiltrates with positive anti-CD-20 (a B-cell marker) and anti-CD-45RO (a T-cell marker) immunoreactivity.^21^ There was negative association with Epstein Barr virus.^21^ The distinction between benign lymphoid hyperplasia and malignant lymphoma necessitated adequate immunohistochemistry and flow cytometry to detect the monoclonal antibodies characteristic of malignant lymphoma. The progression into malignant lymphoma, although rare in conjunctival cases,^21^ has been reported within 1 year and 3.5 years in some case reports.^22^

First, one limitation of our study is the lack of follow-up of the cases in this cohort. Second, a systemic evaluation was not possible due to the retrospective nature of the study and retrieval of data from previous records.

In this study, bulbar lesions were excised mainly from young patients, most of which were nevi. Meanwhile, limbal lesions were excised from older patients, including 18 cases of OSSN and 12 cases of juxtalimbal nevi. The limbal epithelium, being rich in stem cells, was considered a common site for limbal tumors. UV-B or HPV could induce stem cell gene *p*53 (tumor suppressor gene) mutations and transformation into malignancy.^19^,^23^ Hence a lesion that increases in size on the bulbar conjunctival should be considered for biopsy because of a have high incidence of malignant melanoma.^18^ However, most lesions of the caruncle were benign with exception lymphoid hyperplasia that may progress to malignant lymphoma.

In conclusion, this study showed that the most common lesions in an Egyptian cohort were pyogenic granulomas and nevi (mostly compound nevus). Most benign conjunctival tumors were excised at a young age, meanwhile precancerous OSSN was excised in older subjects. The bulbar conjunctiva and limbal area were the most frequent sites of conjunctival tumors, therefore early diagnosis and excision were performed. The absence of truly cancerous lesions suggested their rarity and emphasized the role of early excision of any enlarging lesion in preventing malignant transformation. Results of this study provide a basic source of information on benign conjunctival tumors in an Egyptian population which may be useful for diagnosis and therapy of these tumors, while avoiding overly aggressive treatment for benign or precancerous lesions.

## References

[1] Yanoff M, Sassani JW, Gabbedy R, Nash S (2009). Conjunctiva. Ocular Pathology.

[2] Shields CL, Demirci H, Karatza E, Shields JA (2004). Clinical survey of 1643 melanocytic and nonmelanocytic conjunctival tumors. Ophthalmology.

[3] Mondal SK, Banerjee A, Ghosh A (2007). Histopathological study of conjunctival lesions. J Indian Med Assoc.

[4] Ferry AP (1989). Pyogenic granulomas of the eye and ocular adnexa: A study of 100 cases. Trans Am Ophthalmol Soc.

[5] Espinoza GM, Lueder GT (2005). Conjunctival pyogenic granulomas after strabismus surgery. Ophthalmology.

[6] Panda A, Bhatia IM, Pattnaik NK (1982). Granuloma pyogenicum. Indian J Ophthalmol.

[7] Amoli FA, Heidari AB (2006). Survey of 447 patients with conjunctival neoplastic lesions in Farabi Eye Hospital, Tehran, Iran. Ophthalmic Epidemiol.

[8] Gerner N, Norregaard JC, Jensen OA, Prause JU (1996). Conjunctival naevi in Denmark, 1960-1980: A 21-year follow-up study. Acta Ophthalmol Scand.

[9] Chi MJ, Baek SH (2006). Clinical analysis of benign eyelid and conjunctival tumors. Ophthalmologica.

[10] Folberg R, Jakobiec FA, Bernardino VB, Iwamoto T (1989). Benign conjunctival melanocytic lesions. Clinicopathologic features. Ophthalmology.

[11] Zamir E, Mechoulam H, Micera A, Levi-Schaffer F, Pe’er J (2002). Inflamed juvenile conjunctival naevus: Clinicopathological characterization. Br J Ophthalmol.

[12] Goldenberg-Cohen N, Cohen Y, Rosenbaum E, Herscovici Z, Chowers I, Weinberger D (2005). T1799A BRAF mutations in conjunctival melanocytic lesions. Invest Ophthalmol Vis Sci.

[13] Shields JA, Shields CL, Mashayekhi A, Marr BP, Benavides R, Thangappan A (2008). Primary acquired melanosis of the conjunctiva: Risks for progression to melanoma in 311 eyes. The 2006 Lorenz E. Zimmerman lecture. Ophthalmology.

[14] Lin JY, Fisher DE (2007). Melanocyte biology and skin pigmentation. Nature.

[15] Furdova A, Pesko K, Strmen P, Kobzova M (2007). Conjunctival nevus and melanoma. Bratisl Lek Listy.

[16] Sun EC, Fears TR, Goedert JJ (1997). Epidemiology of squamous cell conjunctival cancer. Cancer Epidemiol Biomarkers Prev.

[17] Basti S, Macsai MS (2003). Ocular surface squamous neoplasia: A review. Cornea.

[18] Shields CL, Shields JA (2004). Tumors of the conjunctiva and cornea. Surv Ophthalmol.

[19] Kiire CA, Dhillon B (2006). The aetiology and associations of conjunctival intraepithelial neoplasia. Br J Ophthalmol.

[20] Bagheri A, Babsharif B, Yazdani S, Rezaee Kanavi M (2007). Benign reactive lymphoid hyperplasia of the caruncle and plica: Report of 5 cases. Ophthal Plast Reconstr Surg.

[21] McLeod SD, Edward DP (1999). Benign lymphoid hyperplasia of the conjunctiva in children. Arch Ophthalmol.

[22] Jenkins C, Rose GE, Bunce C, Cree I, Norton A, Plowman PN (2003). Clinical features associated with survival of patients with lymphoma of the ocular adnexa. Eye (Lond).

[23] Dushku N, Hatcher SL, Albert DM, Reid TW (1999). p53 expression and relation to human papillomavirus infection in pingueculae, pterygia, and limbal tumors. Arch Ophthalmol.

